# The interplay between the disulfide bond formation pathway and cytochrome *c* maturation in *Escherichia coli*

**DOI:** 10.1016/j.febslet.2012.04.055

**Published:** 2012-06-12

**Authors:** Despoina A.I. Mavridou, Stuart J. Ferguson, Julie M. Stevens

**Affiliations:** Department of Biochemistry, University of Oxford, South Parks Road, Oxford OX1 3QU, United Kingdom

**Keywords:** Cytochrome *c*, DsbA, DsbD, Disulfide bond, Cysteine, Heme

## Abstract

Heme attachment to *c*-type cytochromes in bacteria requires cysteine thiols in the CXXCH motif of the protein. The involvement of the periplasmic disulfide generation system in this process remains unclear. We undertake a systematic evaluation of the role of DsbA and DsbD in cytochrome *c* biogenesis in *Escherichia coli* and show unequivocally that DsbA is not essential for holocytochrome production under aerobic or anaerobic conditions. We also prove that DsbD is important but not essential for maturation of *c*-type cytochromes. We discuss the findings in the context of a model in which heme attachment to, and oxidation of, the apocytochrome are competing processes.

## Highlights

► The role of DsbA and DsbD in cytochrome *c* maturation in *E. coli* was probed. ► DsbA is not essential for holocytochrome *c* production. ► DsbD is important but not essential for cytochrome *c* maturation. ► A model is proposed for cytochrome *c* biosynthesis in the periplasm of *E. coli*. ► Heme attachment to and oxidation of the apocytochrome are competing processes.

## 1 Introduction

*c*-Type cytochromes are proteins that contain covalently bound heme and are essential for the life of numerous organisms from all kingdoms of life. In Gram-negative bacteria the heme attachment reaction, typically to the two cysteines of a CXXCH protein motif, is generally performed in the oxidizing environment of the periplasm by the cytochrome *c* maturation (Ccm) system consisting of eight proteins (CcmA–H) [1,2]. Oxidation of the cysteine thiols to a disulfide might reasonably be expected to prevent the heme attachment reaction, creating a chemical paradox that needs to be resolved.

**Fig. 1:**
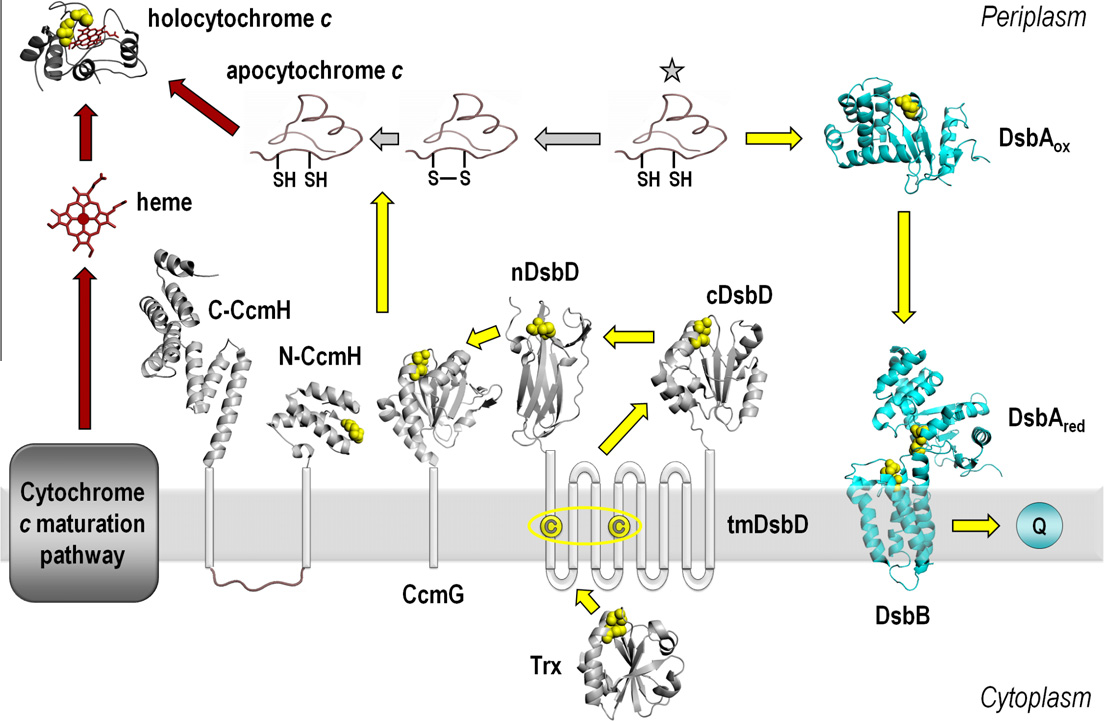
Scheme illustrating the proteins affecting the oxidation state of the CXXCH motif of the apocytochrome before covalent heme attachment in the bacterial periplasm. Apocytochrome enters the periplasm in a reduced state (both cysteines in a thiol form, indicated with a star) and can be oxidized by the protein DsbA which then transfers the acquired electrons to the membrane protein DsbB and itself is restored to its active oxidized state. DsbB passes the electrons to the respiratory chain through quinone (Q). The oxidized apocytochrome (disulfide bond between the cysteines of the CXXCH motif) is reduced by proteins CcmG and/or CcmH of the Ccm system. The necessary reducing power originates from cytoplasmic thioredoxin (Trx). Reductant is transferred sequentially to tmDsbD, cDsbD and nDsbD; the latter is the reductant provider for several periplasmic pathways. The reduced apocytochrome can then have heme attached by the heme-handling proteins CcmA–F. Yellow arrows indicate electron flow and red arrows indicate steps involving heme handling. Cysteines are depicted as yellow spheres. The PDB entries used are 2TRX, 1FVK, 2HI7, 2FWH, 1JPE, 2BLK, 2E2E, and 155C. Structures were rendered in Pymol (DeLano, W.L. The Pymol Molecular Graphics System (2002) http://www.pymol.org).

The periplasmic protein DsbA is crucial for the oxidative folding of extracytoplasmic and extracellular proteins that require disulfide bonds [3] and is linked to the virulence of many pathogens [4]. The characteristics of the modified thioredoxin-like structure of DsbA [5], along with other considerations [6,7], have given insight into its strongly oxidizing properties. DsbB reoxidizes DsbA [8] and transfers electrons to the respiratory chain [9]. The presence of DsbA creates a requirement for proteins with reducing functions in the periplasm to reverse the oxidation reaction and make the apocytochrome thiol groups available for heme attachment. The pathway for reductant provision to the Ccm system as it is currently understood in *Escherichia coli*, along with DsbA and DsbB, are shown in Fig. 1.

DsbD is the sole provider of reducing power to the periplasm. Reductant is needed when incorrectly formed disulfides are inserted into proteins with more than two cysteines, and an isomerase is required, or presumably if *c*-type cytochrome apoproteins acquire disulfide bonds. DsbD is a three-domain protein comprising a transmembrane region with a soluble periplasmic domain at each terminus [10]. It interacts with thioredoxin in the cytoplasm and, via a disulfide cascade [11], transfers reductant to multiple periplasmic partners [12]. DsbC, a dimeric periplasmic disulfide isomerase [13] as well as TrbB, a disulfide bond thioredoxin-like isomerase involved in bacterial conjugation [14] interact with the N-terminal domain of DsbD (nDsbD). For transfer of reductant to the Ccm pathway, nDsbD interacts with the protein CcmG [15]. In the Ccm system CcmG and CcmH (Fig. 1) have been implicated in thiol-oxidoreductase functions. CcmG has a thioredoxin fold but it is not clear whether it interacts with apocytochromes directly, or via thiol–disulfide exchange with CcmH. CcmH has a pair of cysteine residues, in a three-helix bundle fold [16], though the cysteine residues are not essential for cytochrome *c* biogenesis under all growth conditions [17,18].

Several studies have investigated the requirement for DsbA and the involvement of DsbD in cytochrome *c* maturation, with different conclusions. Contrary to intuitive expectation, in *E. coli* strains lacking *dsbA*, the absence of endogenous cytochromes *c* under anaerobic conditions has been reported [19]. The absence of DsbA also resulted in failure to mature an exogenous mono-heme cytochrome *c* [20]. DsbB was found to be essential for cytochrome *c* biogenesis [21], consistent with its role as oxidant of DsbA. These observations were taken as an indication that the formation of disulfide bonds was an obligate step, rather than an undesirable diversion from the heme attachment reaction to apocytochromes. Also, it has been thought that the breakage of the S–S bond could provide a driving force for the formation of the thioether bonds of cytochromes to heme [22]. DsbD was shown to be absolutely essential for cytochrome *c* maturation in *E. coli* [23]. Lack of its analogue, CcdA, was also found to impair cytochrome *c* production in *Rhodobacter capsulatus* [24].

More recently, the failure of DsbA deletion strains to produce cytochromes in *E. coli* was found to vary according to the specific cytochrome involved. A cytochrome *c* from a hyperthermophile was produced at greater than wild-type levels in a Δ*dsbA* strain [25] and a *c*-type variant of cytochrome *b*_562_ was matured under aerobic conditions abundantly and correctly despite the absence of DsbA [26]. Experiments in *R. capsulatus* showed that deletion of *dsbA* did not result in loss of *c*-type cytochromes [27] but that lower cytochrome *c* levels are matured in Δ*dsbA* [24]. It was also shown that in *R. capsulatus* and *Bacillus subtilis* lack of the oxidative proteins (DsdA/DsbB and BdbC/BdbD, respectively) counteracts the cytochrome *c* deficiency in Δ*ccdA* strains [24,27,28]. However, in *E. coli*, the paradigm for study of *c*-type cytochrome biogenesis, such comparative studies with isogenic strains have not been done and thus there are important unresolved contradictions between the original [19–21] and more recent publications.

In this work we have sought to clarify the involvement of DsbA and DsbD in cytochrome *c* biogenesis by examining several endogenous and exogenous cytochromes, with both mono- and multi-heme examples. By using a full set of gene deletion strains and large culture volumes (combined with extracted periplasms being concentrated in small volumes) to ensure that even low levels of cytochrome *c* are detected, we assess the contribution of DsbA and DsbD, both individually and in combination, under aerobic and anaerobic conditions.

## 2 Materials and methods

All bacterial strains and plasmids used in this study are listed in Table 1.

### 2.1 Cell growth

Aerobic cell growth was conducted in 200 ml 2×TY medium (16 g l^−1^ peptone, 10 g l^−1^ yeast extract, 5 g l^−1^ NaCl) in 2.5 l flasks. Cultures were inoculated from single colonies and incubated at 37 °C for 15–18 h with shaking at 200 rpm. Fully aerobic growth conditions prevented expression of the endogenous *E. coli* Ccm system and the Ccm operon was constitutively expressed from plasmid pEC86 [29]. 1 mM isopropyl-1-thio-β-d-galactopyranoside (IPTG) was added to the cultures from inoculation. 100 μg ml^−1^ ampicillin, 20 μg ml^−1^ gentamicin and 34 μg ml^−1^ chloramphenicol were used when appropriate.

Cells were grown anaerobically, allowing the expression of the endogenous Ccm system, for 24 h in 1 l bottles filled to the top with growth media at 37 °C without shaking, inoculated from overnight starter cultures (also grown at 37 °C). Growth media were prepared as described previously [30]. 10 mM nitrate (or 5 mM nitrite in the case of expression of endogenous cytochromes) was used as terminal electron acceptor. 100 μg ml^−1^ ampicillin or 20 μg ml^−1^ gentamicin, were added when appropriate; for the expression of the endogenous *E. coli* cytochromes no antibiotics were used. Cytochrome *c*_550_ was induced with 1 mM IPTG from inoculation and cytochrome *cd*_1_ was induced by autoinduction [31]. When needed, l-cystine (Sigma) was added to the media from inoculation at a final concentration of 5 mM (l-cystine was dissolved in 1 M HCl at a concentration of 5% w/v before addition to the growth media).

### 2.2 Characterization of the cytochrome c content of periplasmic fractions

For extraction of the periplasmic fractions, cells were harvested and sphaeroplasted as described [32]. At least 6 replicates of each experiment were conducted. The production of cytochrome *c*_550_ was quantified by visible absorption spectroscopy on a Varian Cary 50 Bio spectrophotometer. Samples were normalized according to wet-cell pellet weights and were reduced by the addition of a few grains of disodium dithionite (Sigma). Absorbance values at 550 nm were used.

The production of cytochromes *cd*_1_, NapB and NrfA was quantified by SDS–PAGE analysis followed by densitometry on heme-staining bands. SDS–PAGE analysis was carried out on 12% Bis–Tris NuPAGE gels (Invitrogen) using MES–SDS or MOPS–SDS running buffer prepared according to Invitrogen specifications and loading prestained protein markers (Invitrogen, SeeBlue Plus 2). Gels were stained for covalently bound heme according to the method of Goodhew [33]. Gel loadings were normalized according to wet-cell pellet weights. Densitometry was used to quantify cytochrome *c* production using GeneSnap (SYNGENE). The linear relationship between the amount of mature cytochrome *c* present on the gel and the amount detected by densitometry was ensured by using subsaturated loading on gels [34].

Errors on the levels of cytochrome *c* production were calculated for datasets collected on different days. On average these values have a% error of 8%; details of the errors for each set of experiments can be found in Table 2.

## 3 Results and discussion

### 3.1 Cytochrome *c* production under aerobic growth conditions

*E. coli* does not express its endogenous *c*-type cytochromes under aerobic conditions. We used two exogenous cytochromes *c* (cytochromes *c*_550_ and *cd*_1_ from *Paracoccus denitrificans*) as test substrates for the Ccm system in different genetic backgrounds (wild-type, Δ*dsbA*, Δ*dsbD* and Δ*dsbA/ΔdsbD*, see Table 1). Cytochrome *cd*_1_ is produced in its semi-apo form (i.e. without *d*_1_ heme and with heme attached to the *c*-type cytochrome domain) as the *d*_1_ heme cofactor is not produced by *E. coli*. The Ccm proteins were expressed constitutively from plasmid pEC86 [29].

Table 2 shows the quantitation of the levels of production of the two cytochromes as determined spectroscopically (for *c*_550_) and densitometrically (for semi-apo *cd*_1_). The amount of holocytochrome in the wild-type (WT) strain MC1000 is arbitrarily set to 100. For both substrates the heme attachment levels in Δ*dsbA* were very close to those observed in the parental strain. Around 50% levels were detected in the double deletion strain Δ*dsbA/ΔdsbD* in both cases. In the situation where both the oxidizing protein (DsbA) and reducing protein (DsbD) are absent it might be expected that cytochrome levels would approach those seen in the WT strain. However, in the heavily aerated cultures used here, we expect that oxygen would make a significant contribution to spontaneous disulfide bond formation in the periplasm. DsbD would therefore be needed to reduce the proportion of cytochrome that became oxidized via that route. In Δ*dsbD* we found detectable levels of holocytochrome *c*_550_ and significant amounts of holocytochrome *cd*_1._ These observations contrast with previous reports that DsbD is absolutely essential for cytochrome *c* maturation in *E. coli* [35] although the latter study was performed under anaerobic conditions. It has, however, been shown that DsbD is not needed for production of cytochromes *c* in *Pseudomonas aeruginosa* [36] grown aerobically. The absence of DsbD would be expected to attenuate cytochrome levels, as we have observed, because no reductant is being provided to reverse the oxidizing effects of both DsbA and oxygen.

The difference in the relative maturation levels between the two substrate cytochromes is possibly due to their different properties. The accessibility of the pair of cysteine thiols on an apocytochrome delivered to the periplasm will depend on the folding rate of the apoprotein once it is transported by the Sec system. Also, the reduction potential of the thiol–disulfide couple, and the p*K*_a_ values of the cysteine pair of the CXXCH motif, will determine their tendency for oxidation. The potentials could vary dramatically; for example in thioredoxins, which also have a CXXC motif, the character of the XX values, the effect of the helix dipole, and other factors have significant effects on the reduction potentials [37].

### 3.2 Cytochrome *c* production under anaerobic growth conditions

A comparable set of growths to those described above were performed in anaerobic cultures. *E. coli* grown under these conditions produces its endogenous cytochromes *c* [38]; two of these are soluble periplasmic proteins that we have quantitated here, along with the exogenous cytochromes *c*_550_ and *cd*_1_. The endogenous NapB is a di-heme cytochrome *c* involved in nitrate reduction [39] and NrfA is a pentaheme nitrite reductase that has one of its hemes attached to an unusual CXXCK motif [40]. Results are presented in Table 2. There are variations in the heme attachment levels seen for the different cytochromes in the various strains but the trends are the same in all cases. There is a significant decrease in holocytochrome level in Δ*dsbA* compared to WT*,* a similar level when comparing Δ*dsbA* with Δ*dsbA*/*ΔdsbD,* and a reduction in *ΔdsbD*, but not to undetectable levels in any case.

In Δ*dsbA* the significant decrease in holocytochrome level compared to the aerobic cultures highlights the effect of spontaneous disulfide bond formation under aerobic but not under anaerobic conditions. The presence of an oxidant appears to increase holocytochrome levels (as long as a source of reductant (DsbD) is also present) possibly because the disulfide stabilizes the apocytochrome; apocytochromes are readily degraded in the periplasm and are usually not detectable when heme has not been attached to them [41]. It could also be that the oxidized apocytochrome is protected from degradation while interacting with the reducing components of the Ccm system (CcmG or CcmH) and that the most productive holocytochrome biogenesis pathway is one in which the apocytochrome is oxidized, then reduced by the Ccm proteins before heme attachment. To provide evidence for this hypothesis, we measured holocytochrome *c*_550_ levels in anaerobic cultures of Δ*dsbA* supplemented with an oxidant in the growth medium (5 mM l-cystine) and found that it fully complements for the lack of DsbA. For a given set of cultures the amount of holo-*c*_550_ produced in Δ*dsbA* is 39% of what is produced in the parental strain whereas the same strain grown in the presence of 5 mM l-cystine yields 106% holo-*c*_550_. It seems therefore, that DsbA can be replaced not only by oxygen, but by chemical oxidants, in increasing holocytochrome *c* production.

Under anaerobic conditions, similar levels of holocytochrome in Δ*dsbA* and Δ*dsbA/ΔdsbD* are expected. In both cases reduced apocytochrome enters the periplasm and encounters no oxidant (DsbA or oxygen); the presence of DsbD is therefore irrelevant. Hence, the Ccm system attaches heme to the same fraction of apocytochrome in each of the two strains, and the remainder becomes degraded. Again the different properties of the individual cytochromes appear to affect their levels of maturation in the strains examined. These effects are most obvious in the Δ*dsbD* strain (where the level of competition between the Ccm system and DsbA depends on the intrinsic properties of each cytochrome) and the Δ*dsbA/ΔdsbD* strain (where any source of reductant and oxidant, except for spontaneous reoxidation, are removed). More cytochrome *cd*_1_ is produced in Δ*dsbD* (compared to the other cytochromes), both aerobically and anaerobically. Its large size, along with the fact that the protein forms a dimer [42], could protect the CXXCH motif of each monomer from immediate oxidation by DsbA. A larger variation can be seen for the amounts of holocytochromes produced in the Δ*dsbA/ΔdsbD* strain, varying from ∼15% for NrfA to ∼40% for NapB. With any source of oxidant (DsbA, oxygen or chemical) absent, the tendency of the CXXCH motif to find itself in the dead-end situation of disulfide bond formation (since DsbD is absent) could depend on the microenvironment around each motif, which varies for the four substrates (differences in folding rate, number of CXXCH motifs, p*K*_a_ values, and reduction potentials of the cysteine pair being contributing factors).

**Fig. 2:**
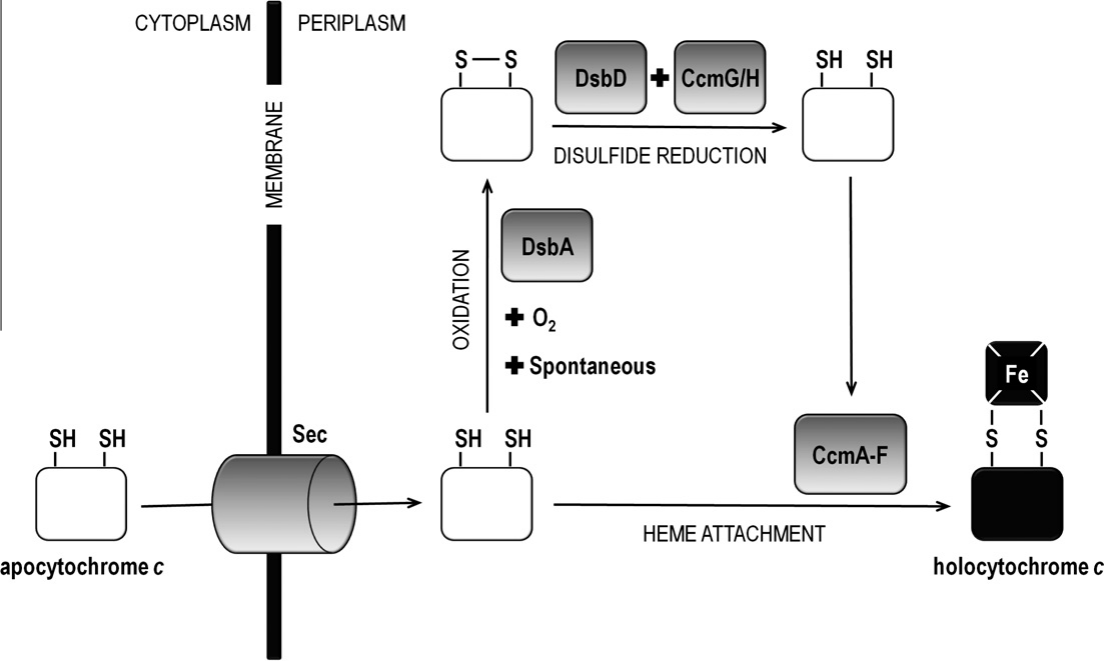
Model illustrating the involvement of DsbA, DsbD and the Ccm system in the formation of holocytochrome *c* in *E. coli*. Upon entering into the periplasm, through the Sec system, the reduced and unfolded apocytochrome can undergo the competing processes of either heme attachment to, or oxidation of, its cysteine thiols. A fraction of the apoprotein interacts with the Ccm heme attachment proteins (CcmA–F) directly. Another fraction instead is oxidized by with DsbA (or oxygen when present). Then it is dependent on the presence of the reducing proteins DsbD and CcmG/H, which eventually deliver it in the reduced state to CcmA–F, for heme attachment. The final product, holocytochrome *c*, can be produced via both pathways.

We propose a model for *E. coli* (shown in Fig. 2) in which the apocytochrome can undergo the competing processes of either heme attachment to, or oxidation of, its cysteine thiols. A proportion of the apoprotein interacts with the Ccm heme attachment proteins directly, involving no thiol–disulfide reactions (for this fraction of apocytochrome DsbD and CcmG/H are irrelevant). Another fraction of the apoprotein instead is oxidized by DsbA (or oxygen when present); the apoform of a CXXCH-containing variant of cytochrome *b*_562_ with a disulfide in its CXXCH motif has been show to be formed [26]. This fraction then is dependent on the presence of the reducing proteins for covalent heme attachment. The ratios of the apocytochrome that will enter each of these pathways will depend on the properties of the individual proteins as illustrated by the varied relative levels of the different substrates in the different strains examined here. It should be taken into account that for multi-heme cytochromes (like NapB and NrfA) it is likely that CXXCH motifs on the same polypeptide chain have different properties. Also attachment of heme to one of the motifs might affect the subsequent heme attachment to the remaining ones. A previous study indicated that a heme binding motif incorporated into a thioredoxin-like fold did not result in high levels of heme attachment [43] demonstrating that the efficiency of heme attachment to CXXCH can be very low depending on the properties of the individual substrate.

### 3.3 Concluding remarks

DsbA is a powerful oxidase that functions outside the cytoplasmic membranes of many bacteria and has a necessarily broad substrate specificity. *c*-Type cytochromes, with their characteristic CXXCH heme-binding motifs, are delivered to the periplasm by the Sec system in their unfolded form [44] and therefore easily become targets for DsbA-catalyzed disulfide-bond formation. The original studies, although a complementation with some small molecule disulfides was seen [20], erroneously concluded that DsbA is essential for holocytochrome *c* production [19–21]. We suspect that the experimental conditions used [19–21] fortuitously led to this conclusion. The present work establishes that the absence of DsbA does have consequences for *c*-type cytochrome formation in *E. coli* for a variety of reasons as explored here which may also relate to comparable observations made in *R. capsulatus*. The Ccm proteins are able to reverse the oxidation event allowing heme attachment to occur; DsbD functions as a house-keeping protein that corrects the unwanted oxidation of apocytochromes. This, along with the potential stabilization of the oxidized apocytochrome, seems to be the most efficient way for these two essential but opposing pathways to coexist in the periplasm but the key point is that mechanistically it is now unambiguous that a disulfide bond is not a prerequisite for heme attachment to a CXXCH motif catalyzed by the Ccm system.

## Figures and Tables

**Table 1:** Bacterial strains and plasmids used in this work.

Name	Description	Source
MC1000	*ara*D139, Δ(*ara*, *leu*)7697, Δ*lac*X74, *gal*U, *gal*K, *str*A	[45]
MC1000 (Δ*dsbA*)	MC1000 Δ*dsbA*::kan	Lab stock
MC1000 (Δ*dsbD*)	MC1000 Δ*dsbD*	[46]
MC1000 (Δ*dsbA*/Δ*dsbD*)	MC1000 Δ*dsbA*::kan, Δ*dsbD*	Lab stock
pEC86	*E. coli ccmABCDEFGH*, Cam^R^	[29]
pRZ001	*P. denitrificans* cytochrome *cd*_1_, Gent^R^	Lab stock
pKPD1	*P. denitrificans c*_550_, Amp^R^	[47]

**Table 2:** Levels of cytochromes *c* produced under aerobic and anaerobic conditions in a set of four bacterial strains. The levels for cytochrome *c*_550_ were determined spectroscopically whereas for cytochromes *cd*_1_, NapB and NrfA SDS–PAGE analysis followed by densitometry on heme-stained gels was used. At least 6 replicates were done for each experiment and errors for each dataset are given in brackets. The amount of cytochrome produced by the wild-type strain is arbitrarily set to 100.

*Aerobic conditions*			
Cytochrome *c*_550_		Cytochrome *cd*_1_	
Strain	Level of cytochrome *c*	Strain	Level of cytochrome *c*
MC1000	100 (±9)	MC1000	100 (±13)
*ΔdsbA*	94 (±2)	*ΔdsbA*	100 (±13)
*ΔdsbD*	6 (±6)	*ΔdsbD*	29 (±10)
*ΔdsbA/ΔdsbD*	53 (±12)	*ΔdsbA/ΔdsbD*	54 (±19)

*Anaerobic conditions*			
Cytochrome *c*_550_		Cytochrome *cd*_1_	

Strain	Level of cytochrome *c*	Strain	Level of cytochrome *c*

MC1000	100 (±8)	MC1000	100 (±8)
*ΔdsbA*	30 (±0)	*ΔdsbA*	39 (±8)
*ΔdsbD*	10 (±5)	*ΔdsbD*	23 (±4)
*ΔdsbA/ΔdsbD*	21 (±19)	*ΔdsbA/ΔdsbD*	31 (±10)

Cytochrome NapB		Cytochrome NrfA	

Strain	Level of cytochrome *c*	Strain	Level of cytochrome *c*

MC1000	100 (±4)	MC1000	100 (±2)
*ΔdsbA*	44 (±7)	*ΔdsbA*	17 (±6)
*ΔdsbD*	4 (±13)	*ΔdsbD*	6 (±3)
*ΔdsbA/ΔdsbD*	39 (±1)	*ΔdsbA/ΔdsbD*	13 (±2)

## References

[1] Kranz R.G., Richard-Fogal C., Taylor J.S., Frawley E.R. (2009). Cytochrome *c* biogenesis: mechanisms for covalent modifications and trafficking of heme and for heme-iron redox control. Microbiol. Mol. Biol. Rev..

[2] Stevens J.M., Mavridou D.A., Hamer R., Kritsiligkou P., Goddard A.D., Ferguson S.J. (2011). Cytochrome c biogenesis System I. FEBS J..

[3] Shouldice S.R., Heras B., Walden P.M., Totsika M., Schembri M.A., Martin J.L. (2011). Structure and function of DsbA, a key bacterial oxidative folding catalyst. Antioxid. Redox. Signal..

[4] Peek J.A., Taylor R.K. (1992). Characterization of a periplasmic thiol:disulfide interchange protein required for the functional maturation of secreted virulence factors of *Vibrio cholerae*. Proc. Natl. Acad. Sci. U S A.

[5] Martin J.L., Bardwell J.C., Kuriyan J. (1993). Crystal structure of the DsbA protein required for disulphide bond formation *in vivo*. Nature.

[6] Grauschopf U., Winther J.R., Korber P., Zander T., Dallinger P., Bardwell J.C. (1995). Why is DsbA such an oxidizing disulfide catalyst?. Cell.

[7] Zapun A., Bardwell J.C., Creighton T.E. (1993). The reactive and destabilizing disulfide bond of DsbA, a protein required for protein disulfide bond formation *in vivo*. Biochemistry.

[8] Inaba K., Murakami S., Nakagawa A., Iida H., Kinjo M., Ito K., Suzuki M. (2009). Dynamic nature of disulphide bond formation catalysts revealed by crystal structures of DsbB. EMBO J..

[9] Inaba K., Ito K. (2008). Structure and mechanisms of the DsbB–DsbA disulfide bond generation machine. Biochim. Biophys. Acta.

[10] Chung J., Chen T., Missiakas D. (2000). Transfer of electrons across the cytoplasmic membrane by DsbD, a membrane protein involved in thiol–disulphide exchange and protein folding in the bacterial periplasm. Mol. Microbiol..

[11] Katzen F., Beckwith J. (2000). Transmembrane electron transfer by the membrane protein DsbD occurs via a disulfide bond cascade. Cell.

[12] Stirnimann C.U., Grutter M.G., Glockshuber R., Capitani G. (2006). NDsbD: a redox interaction hub in the *Escherichia coli* periplasm. Cell. Mol. Life Sci.

[13] Haebel P.W., Goldstone D., Katzen F., Beckwith J., Metcalf P. (2002). The disulfide bond isomerase DsbC is activated by an immunoglobulin-fold thiol oxidoreductase: crystal structure of the DsbC–DsbDalpha complex. EMBO J..

[14] Hemmis C.W., Berkmen M., Eser M., Schildbach J.F. (2011). TrbB from conjugative plasmid F is a structurally distinct disulfide isomerase that requires DsbD for redox state maintenance. J. Bacteriol..

[15] Stirnimann C.U., Rozhkova A., Grauschopf U., Grutter M.G., Glockshuber R., Capitani G. (2005). Structural basis and kinetics of DsbD-dependent cytochrome *c* maturation. Structure.

[16] Di Matteo A., Gianni S., Schinina M.E., Giorgi A., Altieri F., Calosci N., Brunori M., Travaglini-Allocatelli C. (2007). A strategic protein in cytochrome *c* maturation: three-dimensional structure of CcmH and binding to apocytochrome *c*. J. Biol. Chem..

[17] Robertson I.B., Stevens J.M., Ferguson S.J. (2008). Dispensable residues in the active site of the cytochrome *c* biogenesis protein CcmH. FEBS Lett..

[18] Fabianek R.A., Hofer T., Thöny-Meyer L. (1999). Characterization of the *Escherichia coli* CcmH protein reveals new insights into the redox pathway required for cytochrome *c* maturation. Arch. Microbiol..

[19] Metheringham R., Griffiths L., Crooke H., Forsythe S., Cole J. (1995). An essential role for DsbA in cytochrome *c* synthesis and formate-dependent nitrite reduction by *Escherichia coli* K-12. Arch. Microbiol..

[20] Sambongi Y., Ferguson S.J. (1996). Mutants of *Escherichia coli* lacking disulphide oxidoreductases DsbA and DsbB cannot synthesise an exogenous monohaem *c*-type cytochrome except in the presence of disulphide compounds. FEBS Lett..

[21] Metheringham R., Tyson K.L., Crooke H., Missiakas D., Raina S., Cole J.A. (1996). Effects of mutations in genes for proteins involved in disulphide bond formation in the periplasm on the activities of anaerobically induced electron transfer chains in *Escherichia coli* K12. Mol. Gen. Genet..

[22] Moore G.R., Pettigrew G.W. (1990). Cytochrome *c*: evolutionary, structural and physicochemical aspects.

[23] Sambongi Y., Ferguson S.J. (1994). Specific thiol compounds complement deficiency in *c*-type cytochrome biogenesis in *Escherichia coli* carrying a mutation in a membrane-bound disulphide isomerase-like protein. FEBS Lett..

[24] Turkarslan S., Sanders C., Ekici S., Daldal F. (2008). Compensatory thio-redox interactions between DsbA, CcdA and CcmG unveil the apocytochrome *c* holdase role of CcmG during cytochrome *c* maturation. Mol. Microbiol..

[25] Kojima N., Yamanaka M., Ichiki S., Sambongi Y. (2005). Unexpected elevated production of *Aquifex aeolicus* cytochrome *c*_555_ in *Escherichia coli* cells lacking disulfide oxidoreductases. Biosci. Biotechnol. Biochem..

[26] Allen J.W., Barker P.D., Ferguson S.J. (2003). A cytochrome *b*_562_ variant with a *c*-type cytochrome CXXCH heme-binding motif as a probe of the *Escherichia coli* cytochrome *c* maturation system. J. Biol. Chem..

[27] Deshmukh M., Turkarslan S., Astor D., Valkova-Valchanova M., Daldal F. (2003). The dithiol:disulfide oxidoreductases DsbA and DsbB of *Rhodobacter capsulatus* are not directly involved in cytochrome *c* biogenesis, but their inactivation restores the cytochrome *c* biogenesis defect of CcdA-null mutants. J. Bacteriol..

[28] Erlendsson L.S., Hederstedt L. (2002). Mutations in the thiol-disulfide oxidoreductases BdbC and BdbD can suppress cytochrome *c* deficiency of CcdA-defective *Bacillus subtilis* cells. J. Bacteriol..

[29] Arslan E., Schulz H., Zufferey R., Kunzler P., Thöny-Meyer L. (1998). Overproduction of the *Bradyrhizobium japonicum c*-type cytochrome subunits of the *cbb*_3_ oxidase in *Escherichia coli*. Biochem. Biophys. Res. Commun..

[30] Mavridou D.A., Saridakis E., Kritsiligkou P., Goddard A.D., Stevens J.M., Ferguson S.J., Redfield C. (2011). Oxidation state-dependent protein-protein interactions in disulfide cascades. J. Biol. Chem..

[31] Studier F.W. (2005). Protein production by auto-induction in high density shaking cultures. Protein Expr. Purif..

[32] Allen J.W., Tomlinson E.J., Hong L., Ferguson S.J. (2002). The *Escherichia coli* cytochrome *c* maturation (Ccm) system does not detectably attach heme to single cysteine variants of an apocytochrome *c*. J. Biol. Chem..

[33] Goodhew C.F., Brown K.R., Pettigrew G.W. (1986). Haem staining in gels, a useful tool in the study of bacterial *c*-type cytochromes. Biochim. Biophys. Acta.

[34] Goddard A.D., Stevens J.M., Rao F., Mavridou D.A., Chan W., Richardson D.J., Allen J.W., Ferguson S.J. (2010). *C*-Type cytochrome biogenesis can occur via a natural Ccm system lacking CcmH, CcmG, and the heme-binding histidine of CcmE. J. Biol. Chem..

[35] Crooke H., Cole J. (1995). The biogenesis of *c*-type cytochromes in *Escherichia coli* requires a membrane-bound protein, DipZ, with a protein disulphide isomerase-like domain. Mol. Microbiol..

[36] Page M.D., Saunders N.F., Ferguson S.J. (1997). Disruption of the *Pseudomonas aeruginosa dipZ* gene, encoding a putative protein-disulfide reductase, leads to partial pleiotropic deficiency in *c*-type cytochrome biogenesis. Microbiology.

[37] Chivers P.T., Prehoda K.E., Raines R.T. (1997). The CXXC motif: a rheostat in the active site. Biochemistry.

[38] Iobbi-Nivol C., Crooke H., Griffiths L., Grove J., Hussain H., Pommier J., Mejean V., Cole J.A. (1994). A reassessment of the range of *c*-type cytochromes synthesized by *Escherichia coli* K-12. FEMS Microbiol. Lett..

[39] Berks B.C., Richardson D.J., Reilly A., Willis A.C., Ferguson S.J. (1995). The *napEDABC* gene cluster encoding the periplasmic nitrate reductase system of *Thiosphaera pantotropha*. Biochem. J..

[40] Einsle O. (2011). Structure and function of formate-dependent cytochrome *c* nitrite reductase. NrfA. Methods Enzymol..

[41] Gao T., O’Brian M.R. (2007). Control of DegP-dependent degradation of *c*-type cytochromes by heme and the cytochrome *c* maturation system in *Escherichia coli*. J. Bacteriol..

[42] Koppenhofer A., Turner K.L., Allen J.W., Chapman S.K., Ferguson S.J. (2000). Cytochrome *cd*_1_ from *Paracoccus pantotrophus* exhibits kinetically gated, conformationally dependent, highly cooperative two-electron redox behavior. Biochemistry.

[43] Mavridou D.A., Braun M., Thony-Meyer L., Stevens J.M., Ferguson S.J. (2008). Avoidance of the cytochrome *c* biogenesis system by periplasmic CXXCH motifs. Biochem. Soc. Trans..

[44] Thöny-Meyer L., Kunzler P. (1997). Translocation to the periplasm and signal sequence cleavage of preapocytochrome *c* depend on *sec* and *lep*, but not on the *ccm* gene products. Eur. J. Biochem..

[45] Casadaban M.J., Cohen S.N. (1980). Analysis of gene control signals by DNA fusion and cloning in *Escherichia coli*. J. Mol. Biol..

[46] Stewart E.J., Katzen F., Beckwith J. (1999). Six conserved cysteines of the membrane protein DsbD are required for the transfer of electrons from the cytoplasm to the periplasm of *Escherichia coli*. EMBO J..

[47] Sambongi Y., Ferguson S.J. (1994). Synthesis of holo *Paracoccus denitrificans* cytochrome *c*_550_ requires targeting to the periplasm whereas that of holo *Hydrogenobacter thermophilus* cytochrome *c*_552_ does not. Implications for *c*-type cytochrome biogenesis. FEBS Lett..

